# Development of Taiwan Risk Score for Sarcopenia (TRSS) for Sarcopenia Screening among Community-Dwelling Older Adults

**DOI:** 10.3390/ijerph17082859

**Published:** 2020-04-21

**Authors:** Tzyy-Guey Tseng, Chun-Kuan Lu, Yu-Han Hsiao, Shu-Chuan Pan, Chi-Jung Tai, Meng-Chih Lee

**Affiliations:** 1 Department of Family Medicine, Kaohsiung Medical University Hospital, Kaohsiung Medical University, Kaohsiung 80708, Taiwan; tzyyguey@gmail.com; 2 Department of Orthopaedics, Kaohsiung Medical University Hospital, Kaohsiung Medical University, Kaohsiung 80708, Taiwan; u9001054@yahoo.com.tw; 3 Department of Family Medicine, Taichung Hospital, Ministry of Health and Welfare, Taichung 40343, Taiwan; phoebe01026@gmail.com; 4 Department of Public Health, Chung Shan Medical University, Taichung 40201, Taiwan; 5 College of Management, Chaoyang University of Technology, Taichung 41331, Taiwan; 6 Department of Nursing, Pingtung Hospital, Ministry of Health and Welfare, Pingtung 90054, Taiwan; nanapan104@gmail.com; 7 Department of Family Medicine, Pingtung Hospital, Ministry of Health and Welfare, Pingtung 90054, Taiwan; 8 Graduate Institute of Natural Products, College of Pharmacy, Kaohsiung Medical University, Kaohsiung 80708, Taiwan; 9 Institute of Population Health Sciences, National Health Research Institutes, Miaoli 35053, Taiwan

**Keywords:** body composition, handgrip, SARC-F, sarcopenia

## Abstract

The SARC-F questionnaire has been suggested by the European Working Group on Sarcopenia in Older People (EWGSOP) as a first-step screening tool for sarcopenia. However, the sensitivity to SARC-F is low among community-dwelling older adults. Therefore, this study aimed to develop a new prediction model for sarcopenia screening in the community setting. We conducted a cross-sectional analysis of data from the Taiwan Integration of Health and Welfare (TIHW) study. Covariates including comorbidities, socioeconomic status, social support, health behaviors, body composition, and serum biomarkers were collected for analysis. Sarcopenia was defined using handgrip strength and gait speed cut-off values suggested by the Asian Working Group for Sarcopenia. Risk scores for sarcopenia were estimated by stepwise logistic regression. Among 1025 participants (mean age, 71.95 ± 6.89 years), 179 (17.5%) had sarcopenia. Seven items, including age, female sex, receiving social assistance pension, absence of exercise, being underweight, abnormal fasting glucose levels, and abnormal creatinine levels were selected for the Taiwan Risk Scores for Sarcopenia (TRSS) with a cutoff value of 76 (sensitivity, 71.8%; specificity, 71.1%) and area under the curve (AUC) of 0.757. Our results suggested that the TRSS model could be applied cost-effectively in the community for early detection of sarcopenia.

## 1. Introduction

Sarcopenia refers to the loss of lean skeletal muscle mass and diminished muscle strength, which is associated with frailty, disability, mortality, and multiple clinical outcomes among older adults [1]. In long-term clinical outcome, sarcopenia was significantly associated with increased risk of 10-year mortality [2]. Therefore, the early detection of sarcopenia was important for the community-dwelling older adults.

Recently, the revised European Working Group on Sarcopenia in Older People (EWGSOP) algorithm recommended the SARC-F questionnaire as the first step to screen for sarcopenia cases [3]. The SARC-F questionnaire comprises five self-reported components: strength, assistance with walking, rise from a chair, climb stairs, and falls [4]. However, the limitation of using SARC-F in the diagnosis of sarcopenia in community-dwelling older adults was previously studied [5]. First, although the specificity of SARC-F was high (>90%), the sensitivity was very low at 3.8–21.0% [6,7,8]. These results indicated that SARC-F is an effective tool to exclude healthy adults, but sarcopenic candidates selected by SARC-F often needed to undergo further testing to confirm a diagnosis of sarcopenia. Second, Kera and colleagues demonstrated that SARC-F had a higher diagnostic efficacy among older adults with lower physical function than those with higher physical function [5]. The evidence showed that the SARC-F was more suitable for those with older age, those living in nursing homes, and those with a higher number of comorbidities. Third, the diagnostic validity of questionnaire-based SARC-F might be influenced by the cognitive function of older adults. Fourth, a recent study showed that sensitivity (63%) and specificity (47%) of the German version of SARC-F was low for sarcopenia, as defined by reduced hand grip strength and/or impaired chair-rise time [9]. Therefore, the reasons mentioned above indicate that the usability of the SARC-F in a community setting is low.

Barbosa-Silva and colleagues developed SARC-CalF which modified SARC-F by adding calf circumference [10]. They reported that SARC-CalF could significantly increase the sensitivity to 66.7%. Ishii and colleagues developed a screening test using an equation-derived score based on three items: age, grip strength, and calf circumference [11]. Although the sensitivity (75.5–84.9%) and specificity (88.2–92.0%) were high in the Ishii screening test, most general practitioners prefer not to assess grip strength in a clinical setting [12]. Moreover, handgrip strength was recommended by EWGSOP consensus as the second step to assess the probability of sarcopenia, not to find cases.

Importantly, the current recommended strategies for sarcopenia screening prescribe the need for specific training for screening tools. Moreover, it takes time to promote and integrate the strategies into a current health prevention program. For public health, planning separate preventive health policies could result in the double burden of acquiring financial resources for health and welfare. Therefore, the current study aimed to establish a screening model in a different approach for sarcopenia from current evaluation items in health prevention programs in Taiwan.

## 2. Materials and Methods 

### 2.1. Study Participants

In this cross-sectional study, participants were selected from the 2017 Taiwan Health and Welfare (TIHW) study, which was conducted as a formal community screening study for older adults [13]. The participants in the TIHW study were recruited from four aging cities (Pingtung County, Tainan City, Changhua County, and Miaoli County), in which older population had exceeded 14%, and a total of 58 Taiwanese communities were selected. The participants were gathered in their local affiliated community centers for face-to-face interviews and assessments by trained investigators. Before recruitment, all participants received a sufficient explanation about the nature and objective of the study, and provided informed consent for inclusion in the study. The present analysis was restricted to participants aged 60 years and older who completed the questionnaires, physical examination, performance tests, and blood examinations. A total of 1025 participants were included in the current study. The study protocol was approved by the Antai Medical Care Cooperation Antai-Tian-Sheng Memorial Hospital Institutional Review Board in 2017.

### 2.2. Measurements of Physical Functioning

The European Working Group on Sarcopenia recommended using the presence of low muscle mass and low muscle function for the diagnosis of sarcopenia [14]. Low muscle mass indicates probable sarcopenia or dynapenia [15]. However, skeletal muscle loss was not a universal finding in older adults, especially in Asia. In a longitudinal study in Japan, diminished muscle strength was more age dependent than loss of muscle mass [16]. Moreover, Kim and colleagues reported that muscle strength was a better indicator of 5-year adverse clinical outcomes of mortality and low physical performance than muscle mass [17].

The 2014 Asian Working Group for Sarcopenia (AWGS) consensus recommended that handgrip strength less than 26.0 kg in men and 18.0 kg in women were regarded as low muscle strength, and the cutoff value of gait speed was 0.8 m/s [18]. Dominant handgrip strength was evaluated using a hand-held dynamometry and the result is expressed in kilograms (kg). Six meter walk test (6MWT) is a physical performance test used to assess gait speed [18,19]. Therefore, sarcopenia defined by AWGS functional performance criteria was accepted in our study. We agreed with the consensus that simple sarcopenia assessment tools are preferable in community settings [1].

### 2.3. Measures and Biomarkers

In the TIHW study, the items in the questionnaire included participants’ education level, marital status, medical history (hypertension, diabetes, cerebrovascular disease, hyperlipidemia, and heart disease), and socioeconomic status. Socioeconomic status was self-reported, and was not based on the participants’ actual income. Moreover, health behaviors included exercise habits, smoking, and alcohol consumption were also self-reported. The fasting glucose, low-density lipoprotein cholesterol (LDL-C), high-density lipoprotein cholesterol (HDL-C), triglyceride (TG), creatinine level, estimated glomerular filtration rate (eGFR), aspartate aminotransferase (AST), and alanine aminotransferase (ALT) levels of blood tests were provided in of the TIHW study. The blood components examined in this study were included in the annual health examination in Taiwan.

### 2.4. Statistical Analyses

A descriptive analysis of the participants was conducted. We used the T-test to compare the mean values obtained from sarcopenic and non-sarcopenic groups. If two groups had unequal variances, we performed Welch’s test. Bonferroni post-hoc analysis was conducted using MULTTEST procedure in Statistical Analysis Software (SAS). Multivariable regression models were performed on the entire cohort to investigate the predictors of sarcopenia, which were expressed as odds ratios (OR) with 95% confidence interval (CI), adjusted for all covariates. The entire cohort was split, with a 7:3 ratio of training dataset and testing dataset, using the SURVEYSELECT procedure, with simple random sampling method in SAS, followed by a permutation test 2000 times. Moreover, the risk model in prediction of sarcopenia were selected by a stepwise logistic regression using the training dataset. Stepwise selection is a modification of the forward selection, so that after each step in which a variable was added, all candidate variables in the model are checked to see if their significance has been reduced below the specified tolerance level. If a nonsignificant variable is found, it is removed from the model [20]. Using the testing dataset, the receiver operating characteristic (ROC) curve and Youden’s index were used to evaluate the diagnostic accuracy and cutoff values for the prediction model in the determination of sarcopenia. All analyses were conducted using SAS version 9.4 (SAS Institute Inc., Cary, NC, USA).

## 3. Results

### 3.1. Baseline Characteristics

Of the 1,025 enrolled participants, 179 (17.5%) were diagnosed with sarcopenia. Sarcopenic participants were older, and had lower education level, higher self-reported income, higher rate of receiving social assistance pension (9.5% vs. 2.6%), poor exercise habits (55.3% vs. 75.6%), lower alcohol consumption (5.6% vs. 14.3%), and a higher proportion of hypertension and cerebrovascular disease (Table 1). Moreover, sarcopenic participants had higher fasting glucose level (24.4 ± 4.0 vs. 24.9 ± 3.6 mg/dL), lower LDL-C level (109.7 ± 33.5 vs. 116.5 ± 32.6 mg/dL), higher creatinine level (1.04 ± 0.6 vs. 0.92 ± 0.6 mg/dL), and lower eGFR (73.4 ± 22.8 vs. 82.7 ± 22.6 mL/min/1.73 m^2^) (Table 1) [21]. After the adjustment by step-up Bonferroni test, the *p* value of age, educational level, social assistance pension, alcohol consumption, exercise habit, eGFR still showed significance. There was no significant difference in the BMI, AST, ALT, HDL-C, and TG levels between sarcopenic and non-sarcopenic participants.

### 3.2. Predictors of Sarcopenia

Table 2 contains the adjusted odds ratio for each potential risk factor calculated by multivariable logistic regression. In fully adjusted model, being aged above 75 years, receiving social assistance pension, smoking, absence of exercise, abnormal fasting glucose level, and abnormal creatinine level were significantly associated with sarcopenia (Table 2). However, after p value be adjusted based on the number of tests performed, only being aged above 75 years and the absence of exercise were significant predictors. The correlation matrix of all variables was shown in Supplementary Table S1.

### 3.3. Development of Taiwan Risk Scores for Sarcopenia Model

**Figure 1:**
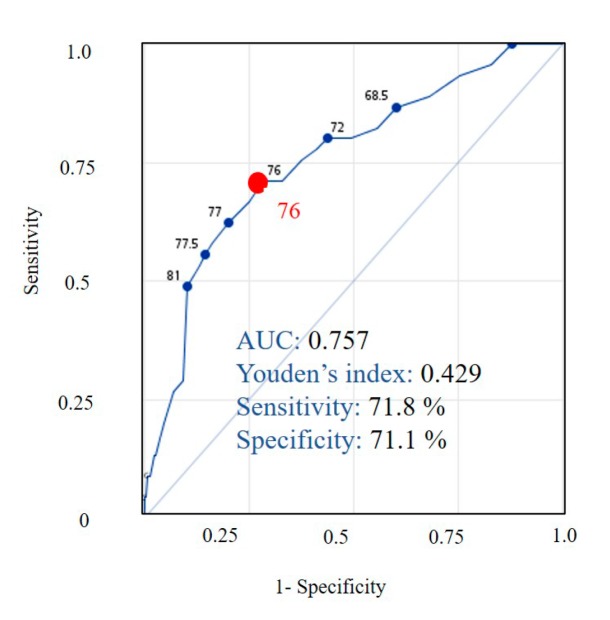
The receiver operating characteristic analysis of the Taiwan Risk Scores for Sarcopenia (TRSS) model. AUC, area under the curve; OR, odds ratio.

By stepwise logistic regression, female, age, receiving social assistance pension, absence of exercise, underweight (BMI < 18.5 kg/m^2^), abnormal fasting glucose level (>126 mg/dL), and abnormal creatinine level (female > 1.2 mg/dL or male > 1.4 mg/dL) were selected as risk scores for sarcopenia from the training dataset (Table 3). The weight of each item was determined by calculating the adjusted odds ratio (adjOR). Risk scores were computed by dividing the individual adjOR by the smallest adjOR in the model and rounding this to the nearest integer. Finally, we developed the Taiwan Risk Scores for Sarcopenia (TRSS) model to predict sarcopenia among community-dwelling older adults. After this, the model was validated on the testing dataset, which showed the cutoff value of 76 (AUC, 0.757; sensitivity, 71.8%; specificity, 71.1%) (Figure 1).

## 4. Discussion

### 4.1. Practical Aspects of TRSS Model for Public Health Policy

In this study, the TRSS model demonstrated that community-dwelling older adults had higher risk of sarcopenia, the sum of risk scores of which was 76 or above. The sensitivity of the TRSS model (71.8%) was significantly higher than SARC-F (3.8–21.0%) and mild higher than SARC-CalF (66.7%) [7,10]. In addition, the AUC (0.757) of the TRSS model was higher than the AUC of SARC-F (0.592–0.667), as reported in the previous study [6,10]. Although the TRSS model could not replace SARC-F and SARC-CalF, it could find sarcopenic cases in a different approach.

Because the seven items in the TRSS model were commonly collected in the annual health exam and the socioeconomic profiles of older adults in Taiwan and many other countries, we proposed that the TRSS model would be an efficient and cost-effective method for the first-line evaluation of sarcopenia in the community. In our opinion, the TRSS model could be initially used to screen sarcopenic cases by the city health bureau, which could provide a target list to medical institutions under its jurisdiction. In the community setting, medical practitioners who are skilled in SARC-F and SARC-CalF could continue to use these screening tools. For those who are not familiar with SARC-F and SARC-CalF, the TRSS could be an alternative screening tool for sarcopenia, while performing an annual health exam among the community-dwelling older adults. As such, we are convinced that the TRSS model can promote and accelerate the detection of sarcopenia.

If the TRSS model were able to identify the older adults with suspected sarcopenia from a previous healthcare database [22], then health policy makers could make a corresponding plan for the target population as early as possible. Since the sensitivity of TRSS is higher than SARC-F, TRSS could reduce the number of sarcopenic candidates requiring further assessment, which would alleviate the burden of general practitioners. Primary care physicians and nursing staff could pay more attention to the assessment and management of sarcopenic older adults. Alternatively, if the TRSS model was applied during the annual health exam in the community, the specificity could be elevated through consecutive screening.

### 4.2. The Associations between Sarcopenia and Items from TRSS Model

The associations between sarcopenia and items from the TRSS model can be explained as follows: a previous study reported that handgrip strength of Caucasians and Asians declines with age [23]. In the Ishii screening test, one extra point is added for every 2 years from 66 years of age for men and two extra points for every 2 years from 66 years of age for women. Similarly, the current study demonstrated that the risk of sarcopenia increases yearly with age, with an adjOR of 1.11. Additionally, subjects with age above 75 years had higher risk of sarcopenia, with an adjOR of 3.44. Age represented the basic score in the TRSS model, and the score also increased year by year. The TRSS model simplified the calculation, compared with the Ishii test. From the public health perspective, according to the TRSS model, we could initially improve healthcare promotion for sarcopenia by focusing on the community-dwelling older adults aged 75 years and above, taking gender into consideration.

The current study demonstrated that females had a higher risk of sarcopenia, with an adjOR of 1.57, compared with males. In a previous study, the sex gap of handgrip strength was higher in the U.S. and Danish populations than in the Japanese population [24]. Therefore, the odds ratio for females may be higher in Western countries. In addition, the prevalence of low handgrip strength was 27.7% in poor older women and 39.6% in poor older men, which were higher than the prevalence in older adults with a normal income [25]. Moreover, high-income older adults had a greater handgrip strength than low-income older adults, especially in women [26]. However, there is no sufficient evidence supporting the association between social determinants and sarcopenia. An ongoing meta-analysis and systematic review has evaluated the relationship between social gradient and sarcopenia [27]. The current study showed that receiving a social assistance pension was a better predictor than self-reported income, marital status, and education level. The weight of receiving social assistance was even higher than gender, exercise habit, or abnormal fasting glucose levels in the TRSS model. These results implied that we should address sarcopenia in older adults who receive a social assistance pension to reduce health inequalities.

Previous studies reported that sarcopenia was associated with lower BMI [28,29,30]. Another study reported that higher BMI with muscle fat infiltration worsens disability [31]. A condition called sarcopenic obesity, in combination with metabolic and functional phenotype in specifically older adults, was introduced [32]. However, the definite cutoff values of BMI in predicting sarcopenia were not established. Hence, Park and colleagues suggested new criteria to diagnose sarcopenia, based on the individual’s appendicular skeletal muscle mass adjusted for body mass index [33]. The current study demonstrated that a BMI lower than 18.5 kg/m^2^ was a significant risk factor of sarcopenia in the TRSS model (Table 3). Further studies are warranted to establish the most appropriate BMI cutoff value predicting sarcopenia, cardiovascular (CV) events, disability, and mortality.

Morley and colleagues reported that the prevalence of sarcopenia was higher in diabetic than in nondiabetic individuals in Korea, India, and the UK [34]. Moreover, a previous study showed that patients with improved blood glucose regulation demonstrated improved physical functions, including gait speed and handgrip strength [35]. Abnormal fasting glucose level (>126 mg/dL) was selected as an independent predictor of sarcopenia in the TRSS model, after adjusting for history of diabetes mellitus and other comorbidities. Therefore, we supposed that the active regulation of blood glucose levels was an effective way to prevent sarcopenia. The role of postprandial sugar level in sarcopenia was not evaluated in the previous and current studies.

In clinical practice, it was not possible to directly measure the GFR in the community setting [36]. Hyun and colleagues demonstrated that the association between sarcopenia alone and eGFR was J-shaped, while the association between sarcopenic obesity and eGFR was U-shaped among adults with a normal BMI [37]. In the current study, sarcopenic participants had significantly lower eGFR level than non-sarcopenic participants. However, a low eGFR of less than 60 mL/min/1.73 m^2^, which would increase mortality, was not an independent predictor of sarcopenia in the current study (Table 2). In contrast, abnormal creatinine level was a significant risk factor in the stepwise logistic regression. This result was similar to those reported in a previous study, in which the sarcopenia index (SI), defined as serum creatinine/serum cystatin C × 100, was an applicable predictor of muscle mass [38]. Moreover, sarcopenic individuals had lower creatinine clearance, which is related to regeneration of muscle tissue [39,40]. Therefore, we supposed that creatinine level might be a better predictor of sarcopenia than eGFR.

### 4.3. Limitations

In interpreting the current study, some limitations should be considered. Firstly, sarcopenia was defined based on physical function and not on muscle mass. Secondly, although we recruited a large number of community-dwelling older adults from aging communities in aging cities, the participants cannot be considered as a representative sample. Therefore, we suggest that the TRSS model should be validated using a national representative cohort in future studies. Thirdly, prospective cohort studies were required to evaluate the effectiveness of active management of common risk factors of CV events and sarcopenia.

## 5. Conclusions

The current study proposed a different approach for sarcopenic screening in the community setting. We developed the TRSS model, which was an age-based prediction model incorporating multidimensional risk factors. The sensitivity, specificity, and AUC of the TRSS model showed that TRSS was an effective tool for screening sarcopenic older adults in the community. The integration of the TRSS model into routine health examinations for the older adults could lead to the early detection of sarcopenic cases and prevent further possible frailty, disability, and mortality.

## Figures and Tables

**Table 1:** Characteristics of the participants (*n* = 1025).

Characteristics	Sarcopenia		Statistics	
	Yes, *n* = 179	No, *n* = 846	Raw *p* Value	Adjusted *p* Value
Age (year), mean ± standard deviation	76.4 ± 7.6	71.0 ± 6.4	<0.001 ^‡^	0.02
Female, *n* (%)	86 (51.5)	629 (54.9)	0.23	0.83
Education, *n* (%)			<0.001 ^†^	0.02
Uneducated	38 (21.2)	82 (9.7)		
Elementary school	84 (46.9)	297 (35.1)		
Junior high school	20 (11.2)	131 (15.5)		
Senior high school	20 (11.2)	191 (22.6)		
College or above	17 (9.5)	145 (17.1)		
Marital status, *n (%)*			0.08	0.72
Married or cohabitant	145 (81.0)	117 (13.8)		
Single, divorced, or widowed	34 (19.0)	729 (86.2)		
Self-reported economic status, *n* (%)			0.047 ^†^	0.55
High-upper income	9 (5.0)	15 (1.8)		
Middle income	83 (46.4)	337 (39.8)		
Low income	87 (48.6)	494 (58.4)		
Social assistance pension, *n* (%)	17 (9.5)	22 (2.6)	<0.001 ^†^	0.02
Smoking, *n* (%)	12 (6.7)	48 (5.7)	0.64	0.83
Alcohol, *n* (%)			<0.001 ^†^	0.02
No	169 (94.4)	725 (85.7)		
Social drinking	6 (3.4)	111 (13.1)		
Alcoholism	4 (2.2)	10 (1.2)		
Exercise habits, *n* (%)			<0.001 ^†^	0.02
No	80 (44.7)	206 (24.4)		
Domestic labor or farming	72 (40.2)	378 (44.7)		
Exercise ≥ 2.5 h/week	27 (15.1)	262 (30.9)		
Self-reported medical history, *n* (%)				
Hypertension	85 (47.5)	320 (37.8)	0.02 ^†^	0.26
Diabetes mellitus	33 (18.4)	120 (14.2)	0.15	0.83
Heart disease	20 (11.2)	65 (7.7)	0.12	0.83
Hyperlipidemia	4 (2.2)	49 (5.8)	0.05	0.55
Cerebrovascular disease	11 (6.2)	18 (2.1)	0.003 ^†^	0.05
Physical and blood examinations, mean ± standard deviation				
BMI, kg/m^2^	24.4 ± 4.0	24.9 ± 3.6	0.17	0.83
Fasting glucose level, mg/dL	110.6 ± 41.3	101.5 ± 22.6	0.005 ^‡^	0.08
Aspartate aminotransferase, IU/L	26.0 ± 14.3	25.2 ± 10.8	0.47	0.83
Alanine aminotransferase, IU/L	21.0 ± 17.0	23.6 ± 15.0	0.06	0.60
Low-density lipoprotein cholesterol, mg/dL	109.7 ± 33.5	116.5 ± 32.6	0.01 *	0.14
High-density lipoprotein cholesterol, mg/dL	57.9 ± 16.8	57.5 ± 16.9	0.80	0.83
Triglyceride, mg/dL	118.0 ± 66.9	116.7 ± 73.4	0.83	0.83
Creatinine, mg/dL	1.04 ± 0.60	0.92 ± 0.55	0.01 *	0.14
Estimated glomerular filtration rate, mL/min/1.73 m^2^	73.4 ± 22.8	82.7 ± 22.6	< 0.001 *	0.02

* T-test, *p* < 0.05; ^†^ Chi-square test, *p* < 0.05; ^‡^ Welch’s test, *p* < 0.05; Adjusted *p*-values were estimated by step-up Bonferroni adjustment.

**Table 2:** Multivariable logistic regression model in prediction of sarcopenia.

Variables	Adjusted OR (95% CI)	Raw *p* Value	Adjusted *p* Value
Female	1.41 (0.94–2.11)	0.10	0.88
Age (75 < age)	3.44 (2.36–5.02)	<0.001 *	0.02 *
Education (elementary school or below)	1.36 (0.82–2.24)	0.23	0.88
Single, divorced or widowed	1.15 (0.71–1.86)	0.56	0.88
Self-reported low income	1.61 (0.60–4.33)	0.35	0.88
Social assistance pension	3.04 (1.35–6.84)	0.007 *	0.13
Underweight (BMI < 18.5 kg/m^2^)	2.31 (1.01–5.33)	0.05 *	0.75
Smoking	1.85 (1.01–3.40)	0.05 *	0.75
Alcohol	0.61 (0.33–1.13)	0.11	0.88
Absence of exercise	2.28 (1.57–3.30)	<0.001 *	0.02 *
Self-reported medical history			
Heart disease	0.95 (0.51–1.78)	0.88	0.88
Hypertension	1.17 (0.81–1.70)	0.40	0.88
Diabetes mellitus	0.77 (0.43–1.37)	0.37	0.88
Cerebrovascular disease	2.36 (0.93–5.98)	0.07	0.88
Hyperlipidemia	0.48 (0.16–1.50)	0.20	0.88
Blood examination evaluation			
Fasting glucose level (>126 mg/dL)	2.24 (1.25–4.00)	0.007 *	0.13
Elevated LDL-C level (>130 mg/dL)	0.77 (0.51–1.17)	0.23	0.88
Low HDL-C level (<40 mg/dL)	0.86 (0.49–1.52)	0.61	0.88
Elevated Triglyceride (>150 mg/dL)	1.04 (0.67–1.63)	0.86	0.88
Abnormal creatinine level ^†^	1.97 (0.99–3.89)	0.05 *	0.75
eGFR (<60 mL/min/1.73 m^2^)	1.10 (0.80–1.52)	0.55	0.88

Odds ratio was adjusted for covariates listed in Table 1. * Multivariable logistic regression, *p* < 0.05. Adjusted *p*-values were estimated by step-up Bonferroni adjustment. ^†^ Abnormal creatinine level: female > 1.2 mg/dL and male > 1.4 mg/dL. BMI, body mass index; CI, confidence interval; eGFR, estimated glomerular filtration rate; HDL-C, high-density lipoprotein cholesterol; LDL-C, low-density lipoprotein cholesterol; OR, odds ratio.

**Table 3:** Taiwan Risk Scores for Sarcopenia (TRSS) Model Estimated by Stepwise Logistic Regression.

Variables	Adjusted OR (95% CI)	Risk Score Weights
Age	1.11 (1.08–1.15)	1
Female	1.53 (1.01–2.35)	1
Social assistance pension	3.90 (1.66–9.15)	4
Absence of exercise	2.30 (1.50–3.50)	2
Underweight ^†^	3.07 (1.06–8.87)	3
Abnormal fasting glucose level ^‡^	2.17 (1.22–3.88)	2
Abnormal creatinine level ^§^	2.26 (1.15–4.47)	2

^†^ Underweight: body mass index <18.5 kg/m^2^; ^‡^ Fasting glucose level >126 mg/dL; ^§^ Creatinine level: female > 1.2 mg/dL or male > 1.4 mg/dL. Risk scores were computed by dividing the individual adjusted OR by the smallest adjusted OR in the model and rounding this to the nearest integer. The sum of age and the other risk scores in the TRSS model was the total score of each individual. OR, odds ratio.

## Supplementary Materials

The following are available online at https://www.mdpi.com/1660-4601/17/8/2859/s1, Table S1: Correlation matrix of all variables listed in Table 2.
