# Supplementary material for: Development of Taiwan Risk Score for Sarcopenia (TRSS) for Sarcopenia Screening among Community-Dwelling Older Adults
Source: Int J Environ Res Public Health. 2020 Apr 21;17(8):2859. doi: 10.3390/ijerph17082859 (PMC7216229; doi:10.3390/ijerph17082859)
Supplement: Supplementary file 1 [file ijerph-17-02859-s001.pdf]

Supplementary material

Table S1. Correlation matrix of variables listed in Table 2.

Pearson Correlation Coefficients, N = 1025

Prob > |r| under H0: Rho=0

	**female**	**age4**	**low_edu**	**marital_stat**	**low_income**	**pension**	**uw**	**cigar**	**alcohol**	**no_sport**	**ht**	**dm**	**hd**	**lip**	**cva**	**ifg**	**ab_ldl**	**ab_cr**	**ab_egfr**	**ab_hdl**	**ab_TG**
**female**	1	-0.05396	0.16115	0.09124	-0.02737	-0.05436	0.02836	-0.25142	-0.29072	0.03385	0.01897	0.00225	-0.02444	-0.06156	-0.03361	-0.04539	0.08786	-0.12022	-0.16746	-0.18929	0.04828
		0.0842	<.0001	0.0035	0.3813	0.0819	0.3643	<.0001	<.0001	0.279	0.5441	0.9426	0.4345	0.0488	0.2823	0.1464	0.0049	0.0001	<.0001	<.0001	0.1224
**age4**	-0.05396	1	0.20973	0.08721	0.03508	0.05709	0.05635	-0.10021	-0.0996	0.07218	0.11358	0.00811	0.11294	-0.07908	0.0474	0.05251	-0.0633	0.15496	0.20244	0.01342	-0.02034
	0.0842		<.0001	0.0052	0.2618	0.0677	0.0713	0.0013	0.0014	0.0208	0.0003	0.7954	0.0003	0.0113	0.1294	0.0929	0.0427	<.0001	<.0001	0.6679	0.5153
**low_edu**	0.16115	0.20973	1	0.02844	0.02388	0.07033	0.02187	-0.08551	-0.07848	0.09822	0.07812	0.03481	0.08857	-0.05762	0.01107	0.07387	-0.0098	0.02586	0.00236	0.02767	-0.00079
	<.0001	<.0001		0.363	0.4449	0.0243	0.4842	0.0062	0.012	0.0016	0.0124	0.2655	0.0045	0.0652	0.7233	0.018	0.7541	0.4082	0.9399	0.3761	0.9799
**marital_stat**	0.09124	0.08721	0.02844	1	0.11768	0.03244	0.05201	0.0084	0.00456	-0.00081	0.0019	-0.01962	0.0447	-0.05977	-0.02112	-0.01571	-0.02194	0.02062	-0.01172	-0.00713	0.00766
	0.0035	0.0052	0.363		0.0002	0.2995	0.0961	0.7881	0.8841	0.9792	0.9517	0.5304	0.1527	0.0558	0.4994	0.6154	0.4829	0.5095	0.7079	0.8196	0.8065
**low_income**	-0.0273	0.03508	0.02388	0.11768	1	0.30644	0.1206	-0.0156	-0.0233	0.03313	0.05961	0.04377	0.04702	-0.0070	-0.0264	-0.0137	-0.0296	0.08037	0.00705	0.00006	-0.0034

	7							6	4					2	2	3	2				6
	0.3813	0.2618	0.4449	0.0002		<.0001	0.0001	0.6166	0.4555	0.2892	0.0564	0.1614	0.1325	0.8224	0.3981	0.6605	0.3435	0.0101	0.8216	0.9985	0.9118
**pension**	-0.05436	0.05709	0.07033	0.03244	0.30644	1	-0.00638	-0.03034	-0.00684	0.04682	0.03745	0.0598	0.03265	-0.00038	0.02758	0.01074	-0.03944	0.12036	0.07182	0.01743	0.01873
	0.0819	0.0677	0.0243	0.2995	<.0001		0.8384	0.3319	0.827	0.1341	0.2309	0.0556	0.2963	0.9903	0.3778	0.7312	0.2071	0.0001	0.0215	0.5773	0.5493
**uw**	0.02836	0.05635	0.02187	0.05201	0.1206	-0.00638	1	-0.00615	-0.06584	-0.03663	-0.08771	-0.04371	0.04773	-0.04192	-0.03063	-0.02782	-0.04253	0.03573	0.00818	-0.06781	-0.06747
	0.3643	0.0713	0.4842	0.0961	0.0001	0.8384		0.8442	0.0351	0.2413	0.005	0.162	0.1267	0.1799	0.3272	0.3736	0.1736	0.2531	0.7937	0.0299	0.0308
**cigar**	-0.25142	-0.10021	-0.08551	0.0084	-0.01566	-0.03034	-0.00615	1	0.27918	0.01447	-0.01047	0.01586	-0.00249	-0.01242	-0.02119	0.04639	0.01255	0.04223	0.04679	0.02495	-0.00241
	<.0001	0.0013	0.0062	0.7881	0.6166	0.3319	0.8442		<.0001	0.6435	0.7379	0.6121	0.9365	0.6913	0.498	0.1377	0.6881	0.1767	0.1344	0.4249	0.9387
**alcohol**	-0.29072	-0.0996	-0.07848	0.00456	-0.02334	-0.00684	-0.06584	0.27918	1	-0.03078	-0.05843	-0.05426	-0.02774	0.05143	-0.06258	0.02312	-0.01033	0.00379	-0.00652	0.01448	0.01504
	<.0001	0.0014	0.012	0.8841	0.4555	0.827	0.0351	<.0001		0.3248	0.0615	0.0825	0.375	0.0998	0.0452	0.4598	0.7412	0.9035	0.8349	0.6433	0.6305
**no_sport**	0.03385	0.07218	0.09822	-0.00081	0.03313	0.04682	-0.03663	0.01447	-0.03078	1	0.01333	0.0263	-0.02143	-0.04704	0.09063	0.01516	0.02601	0.07579	0.06828	0.08741	-0.00117
	0.279	0.0208	0.0016	0.9792	0.2892	0.1341	0.2413	0.6435	0.3248		0.67	0.4002	0.4931	0.1324	0.0037	0.6278	0.4056	0.0152	0.0288	0.0051	0.9701
**ht**	0.01897	0.11358	0.07812	0.0019	0.05961	0.03745	-0.08771	-0.01047	-0.05843	0.01333	1	0.19346	0.02471	0.08164	0.07873	0.11397	-0.10374	0.07177	0.09179	0.08104	0.04783

	0.5441	0.0003	0.0124	0.9517	0.0564	0.2309	0.005	0.7379	0.0615	0.67		<.0001	0.4294	0.0089	0.0117	0.0003	0.0009	0.0216	0.0033	0.0094	0.126
**dm**	0.00225	0.00811	0.03481	-0.01962	0.04377	0.0598	-0.04371	0.01586	-0.05426	0.0263	0.19346	1	0.05274	0.18655	0.02759	0.46137	-0.15736	0.18718	0.09235	0.12334	0.01137
	0.9426	0.7954	0.2655	0.5304	0.1614	0.0556	0.162	0.6121	0.0825	0.4002	<.0001		0.0915	<.0001	0.3775	<.0001	<.0001	<.0001	0.0031	<.0001	0.7162
**hd**	-0.02444	0.11294	0.08857	0.0447	0.04702	0.03265	0.04773	-0.00249	-0.02774	-0.02143	0.02471	0.05274	1	0.07357	0.09804	0.00615	-0.08626	0.14645	0.11115	0.03623	-0.02137
	0.4345	0.0003	0.0045	0.1527	0.1325	0.2963	0.1267	0.9365	0.375	0.4931	0.4294	0.0915		0.0185	0.0017	0.8442	0.0057	<.0001	0.0004	0.2465	0.4943
**lip**	-0.06156	-0.07908	-0.05762	-0.05977	-0.00702	-0.00038	-0.04192	-0.01242	0.05143	-0.04704	0.08164	0.18655	0.07357	1	0.03987	0.05753	-0.04506	0.01898	-0.0126	0.07172	0.07976
	0.0488	0.0113	0.0652	0.0558	0.8224	0.9903	0.1799	0.6913	0.0998	0.1324	0.0089	<.0001	0.0185		0.2022	0.0656	0.1494	0.5438	0.6869	0.0217	0.0106
**cva**	-0.03361	0.0474	0.01107	-0.02112	-0.02642	0.02758	-0.03063	-0.02119	-0.06258	0.09063	0.07873	0.02759	0.09804	0.03987	1	-0.00422	-0.07186	0.04243	0.00432	0.06013	0.00985
	0.2823	0.1294	0.7233	0.4994	0.3981	0.3778	0.3272	0.498	0.0452	0.0037	0.0117	0.3775	0.0017	0.2022		0.8927	0.0214	0.1746	0.89	0.0543	0.7528
**ifg**	-0.04539	0.05251	0.07387	-0.01571	-0.01373	0.01074	-0.02782	0.04639	0.02312	0.01516	0.11397	0.46137	0.00615	0.05753	-0.00422	1	-0.08636	0.18658	0.10462	0.08226	0.06918
	0.1464	0.0929	0.018	0.6154	0.6605	0.7312	0.3736	0.1377	0.4598	0.6278	0.0003	<.0001	0.8442	0.0656	0.8927		0.0057	<.0001	0.0008	0.0084	0.0268
**ab_ldl**	0.08786	-0.0633	-0.0098	-0.02194	-0.02962	-0.03944	-0.04253	0.01255	-0.01033	0.02601	-0.10374	-0.15736	-0.08626	-0.04506	-0.07186	-0.08636	1	-0.0835	-0.10791	-0.09596	0.11959
	0.0049	0.0427	0.7541	0.4829	0.3435	0.2071	0.1736	0.6881	0.7412	0.4056	0.0009	<.0001	0.0057	0.1494	0.0214	0.0057		0.0075	0.0005	0.0021	0.0001

**ab_cr**	-0.12022	0.15496	0.02586	0.02062	0.08037	0.12036	0.03573	0.04223	0.00379	0.07579	0.07177	0.18718	0.14645	0.01898	0.04243	0.18658	-0.0835	1	0.47169	0.10918	-0.00288
	0.0001	<.0001	0.4082	0.5095	0.0101	0.0001	0.2531	0.1767	0.9035	0.0152	0.0216	<.0001	<.0001	0.5438	0.1746	<.0001	0.0075		<.0001	0.0005	0.9266
**ab_egfr**	-0.16746	0.20244	0.00236	-0.01172	0.00705	0.07182	0.00818	0.04679	-0.00652	0.06828	0.09179	0.09235	0.11115	-0.0126	0.00432	0.10462	-0.10791	0.47169	1	0.10484	0.03834
	<.0001	<.0001	0.9399	0.7079	0.8216	0.0215	0.7937	0.1344	0.8349	0.0288	0.0033	0.0031	0.0004	0.6869	0.89	0.0008	0.0005	<.0001		0.0008	0.22
**ab_hdl**	-0.18929	0.01342	0.02767	-0.00713	0.00006	0.01743	-0.06781	0.02495	0.01448	0.08741	0.08104	0.12334	0.03623	0.07172	0.06013	0.08226	-0.09596	0.10918	0.10484	1	0.19422
	<.0001	0.6679	0.3761	0.8196	0.9985	0.5773	0.0299	0.4249	0.6433	0.0051	0.0094	<.0001	0.2465	0.0217	0.0543	0.0084	0.0021	0.0005	0.0008		<.0001
**ab_TG**	0.04828	-0.02034	-0.00079	0.00766	-0.00346	0.01873	-0.06747	-0.00241	0.01504	-0.00117	0.04783	0.01137	-0.02137	0.07976	0.00985	0.06918	0.11959	-0.00288	0.03834	0.19422	1
	0.1224	0.5153	0.9799	0.8065	0.9118	0.5493	0.0308	0.9387	0.6305	0.9701	0.126	0.7162	0.4943	0.0106	0.7528	0.0268	0.0001	0.9266	0.22	<.0001	
